# Synergistic effect of ligand–cluster structure and support in gold nanocluster catalysts for selective hydrogenation of alkynes†

**DOI:** 10.1039/d4nr03865g

**Published:** 2025-01-14

**Authors:** Rareṣ Banu, Adea Loxha, Nicole Müller, Stylianos Spyroglou, Egon Erwin Rosenberg, A. Eduardo Palomares, Fernando Rey, Carlo Marini, Noelia Barrabés

**Affiliations:** a Institue of Materials Chemistry, TU Wien Getreidemarkt 9/E165 1060 Vienna Austria noelia.rabanal@tuwien.ac.at; b Institute of Chemical, Environmental and Bioscience Engineering, TU Wien Getreidemarkt 9/E166 1060 Vienna Austria; c Institute of Chemical Technologies and Analytics, TU Wien Getreidemarkt 9/E164 1060 Vienna Austria; d Instituto de Tecnología Química, Universitat Politécnica de Valéncia – Consejo Superior de Investigaciones Científicas (UPV-CSIC) Valencia Spain; e ALBA Synchrotron Light Facility Carrer de la Llum 2-26 08290 Cerdanyola del Valles Barcelona Spain

## Abstract

In the field of nanocluster catalysis, it is crucial to understand the interplay of different parameters, such as ligands, support and pretreatment and their effect on the catalytic process. In this study, we chose the selective hydrogenation of phenylacetylene as a model reaction and employed two gold nanoclusters as catalysts, the phosphine protected Au_11_ and the thiolate protected Au_25_, each with different binding motifs. They were supported on MgO, Al_2_O_3_ and a hydrotalcite (HT), chosen for their different acidity. We found that while in the case of the Au_11_ MgO was the preferred support and the pretreatment had a positive impact on the catalysis. For the Au_25_ the HT performed best and pretreating had negative effects, indicating that the bonding motif of the ligands and their interaction with the support is crucial for the catalytic process. Using X-ray absorption spectroscopy we could trace these phenomena to changes in the cluster–ligand interface, which seem to impact the stability of the catalysts.

## Abstract 2

Disclosing the effect of ligand binding motif types on the catalytic activity of gold nanoclusters in selective hydrogenation.

## Graphical abstract

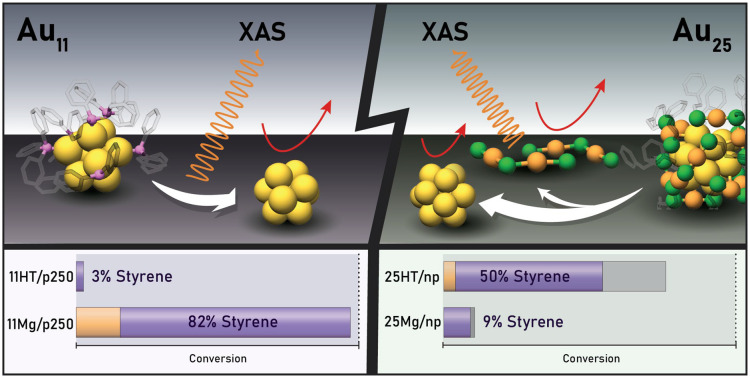

## 1 Introduction

Alkynes, characterized by their carbon–carbon triple bonds, are significant byproducts in various industrial processes, particularly within the oil sector. Their unique structural properties endow them with substantial synthetic potential, making them valuable in the production of a wide array of chemicals, including pharmaceuticals, fine chemicals, and natural products. The conversion of alkynes to alkenes is particularly crucial, as alkenes serve as versatile building blocks in these applications. However, achieving selective hydrogenation of alkynes to alkenes poses considerable challenges, primarily due to the tendency of catalysts to promote unwanted side reactions.^1–6^

Palladium-based catalysts are commonly employed for this hydrogenation process, owing to their effectiveness in facilitating the conversion. Nevertheless, these catalysts can lead to undesired outcomes, such as complete hydrogenation to alkanes or oligomerization, which results in the accumulation of heavier hydrocarbons on the catalyst surface. This not only diminishes the efficiency of the reaction but also accelerates catalyst deactivation, necessitating further optimization of catalytic systems.^3,5,7,8^

A classic solution to gain control over the reaction is to poison the catalyst with Pb and quinoline, resulting in the so-called Lindlar catalyst.^1,3^ While this catalyst offers several advantages, such as its heterogeneous nature, ease of synthesis and improved selectivity, it also has notable drawbacks, including Pb toxicity, lack of selectivity for some alkynes and problems with reproducibility.^1^ It is therefore necessary to look for alternatives.

One approach that has been explored is the incorporation of various metals such as Ru, Ag, Au, Cu, Pb and In to modulate the atomic and electronic structure of the surface. These metals can act as promoters or be used in bimetallic catalysts to enhance activity and selectivity. Previous reported studies have highlighted the remarkable performance of supported gold catalysts, such as Au/Al_2_O_3_, Au/TiO_2_ and Au/Fe_2_O_3_, in the hydrogenation of acetylene and propyne.^9–11^ These catalysts exhibit exceptional selectivity compared to other monometallic systems, showing intrinsic selectivity for triple bond hydrogenation due to its preferential alkyne adsorption.^11^

Strategies involving collaboration between coordinatively unsaturated Au atoms and acid–base pairs on supports have been proposed to enhance H_2_ dissociation and improve catalytic properties.^12,13^ This concept was first explored by Nikolaev and Smirnov,^14^ who investigated gold nanoparticles for the heterogeneous semihydrogenation of phenylacetylene (PA). Their study revealed that Au nanoparticles supported on Al_2_O_3_ exhibited remarkable activity, selectivity, and stability. Crucially, they found that smaller particle sizes enhanced catalytic performance, attributing this to an increase in surface and corner sites, as well as a decrease in electron density around the particle surface due to interactions with the alumina support. Therefore, it was shown that the critical interplay between catalyst structure, particularly particle size, and the support material influence the electronic properties and morphology of the active metal nanoparticles.^12^

G.C. Bond^15^ showed how small gold particles supported on basic oxides like MgO enhance hydrogenation reactions by facilitating hydrogen dissociation and chemisorption. The basic nature of MgO complements the gold catalyst by providing basic sites that assist in the adsorption and activation of hydrogen and other reactants. This synergistic interaction between the gold nanoparticles and the MgO support modifies the electronic properties of gold, improving its catalytic performance. Overall, this combination results in a more effective catalyst system, demonstrating the benefits of engineering both the active metal and its support for enhanced activity and selectivity in hydrogenation reactions.

Another approach at controlling the hydrogen activation and reactivity of gold based catalysts is the use of ligands, which have significant effects on the hydrogenation of alkynes and the activation of hydrogen. With regard to hydrogen activation, specific ligands can facilitate the heterolytic activation of H_2_. In the case of gold–ligand catalysed selective hydrogenation, the interaction between gold and the ligands can create frustrated Lewis pairs (FLPs) which allow the heterolytic cleavage of H_2_ into a proton (H^+^) and a hydride (H^−^). This tightly bound ion pair can then be selectively transferred to an alkyne, leading to the formation of *cis*-alkenes. This process is controlled by electrostatic interactions between the ion pair and the alkyne.^12,16–19^

In this context, atomically precise monolayer-protected gold nanoclusters have emerged as promising active sites for selective hydrogenation.^20–24^ In particular, gold clusters protected by alkynyl ligands ([Au_38_(PhC≡C)_20_(Ph3P)_4_]^2+^) exhibit high activity in the semihydrogenation of alkynes to alkenes, whereas those protected by thiolate ligands (Au_38_(3-methylbenzenethiol)_20_(Ph3P)_4_) show very low activity under the same conditions.^24^ This suggests that the type of ligand significantly influences catalytic activity by altering the electronic structure of the gold nanoclusters.

In our previous studies on gold nanocluster catalysts for the selective hydrogenation of phenylacetylene, we observed a strong effect of the acid–base properties of the oxide support material.^25^ Therein, the effect of strong pretreatment conditions, which removed the ligand shell, was evaluated.

In this work, we focus on understanding the different effects between using thiolate-protected Au_25_ and phosphine-protected Au_11_ nanoclusters, concentrating on the ligand effect and ligand–support interactions. Three different metal oxides have been employed as support: Al_2_O_3_, MgO, and hydrotalcite. Each material, with distinct acid–base properties, influences the stabilization, the interaction with the ligand shell and reactivity of the clusters. Therefore, by using structural analysis by X-ray Absorption Spectroscopy (XAFS), we correlate the effects of these variables on the selective hydrogenation of phenylacetylene to styrene (ST).

## 2 Results and discussion

**Fig. 1:**
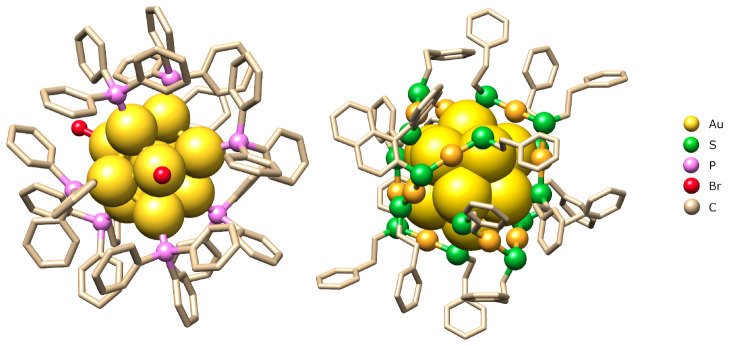
Structure of the synthesized nanoclusters.

The support, ligand, and pretreatment effects on gold nanoclusters’ activity and selectivity were investigated for the liquid-phase semihydrogenation of phenylacetylene. Two types of gold nanoclusters were synthesized and characterized: Au_11_(PPh_3_)_7_Br_3_ and Au_25_(SC_2_H_4_Ph)_18_. Thiolate-protected Au_25_ and phosphine-protected Au_11_ nanoclusters exhibit notable differences in structure, charge distribution, ligand–metal interaction, stability, and electronic properties. Au_25_(SR)_18_ features a complex core–shell structure with an icosahedral Au_13_ core and six –RS–Au–SR–Au–SR– staple motifs, resulting in a highly stable, larger structure with strong covalent Au–S bonds and discrete electronic states. In contrast, Au_11_(PPh_3_)_7_Br_3_ has a simpler and more compact structure with directly bound phosphine ligands and weaker coordination bonds, making it less stable and more prone to transformation into larger nanoparticles. The differences in charge distribution, with Au_25_(SR)_18_ typically carrying a negative charge on the staple Au, and Au_11_(PPh_3_)_7_Br_3_ a positive charge, along with distinct electronic properties, influence their stability and reactivity.^26^ The structures of these aforementioned nanoclusters are presented in Fig. 1.

To examine the support effects, the nanoclusters were deposited on three different metal oxides: Al_2_O_3_ (acidic), MgO (basic), and hydrotalcite (HT, layered oxide composed of Al_2_O_3_ and MgO, with an intermediate basicity). Details on the preparation and characterization of nanocluster catalysts can be found in the ESI.†

Previous works have shown that the pretreatment of such catalysts can have a significant effect on their catalytic performance, due to the ensuing of temperature dependent ligand removal.^27^ To explore this effect, the catalysts were investigated under different pretreatment conditions, including non-pretreatment, calcination at 150 °C and 250 °C in oxidative atmosphere (air) (details in ESI†), leading to different ligand shell presence. Lower temperatures led to partial ligand removal, while higher temperatures caused more extensive removal, being also affected by the type of support employed.^25,28^ This resulted in a total of six different catalysts, each with two additional pretreated versions, which were used in the liquid phase hydrogenation of phenylacetylene. For clarity, the catalysts will be referred to in the following discussion as 11Al, 11Mg, 11HT, 25Al, 25Mg and 25HT.

### 2.1 Catalytic activity

**Fig. 2:**
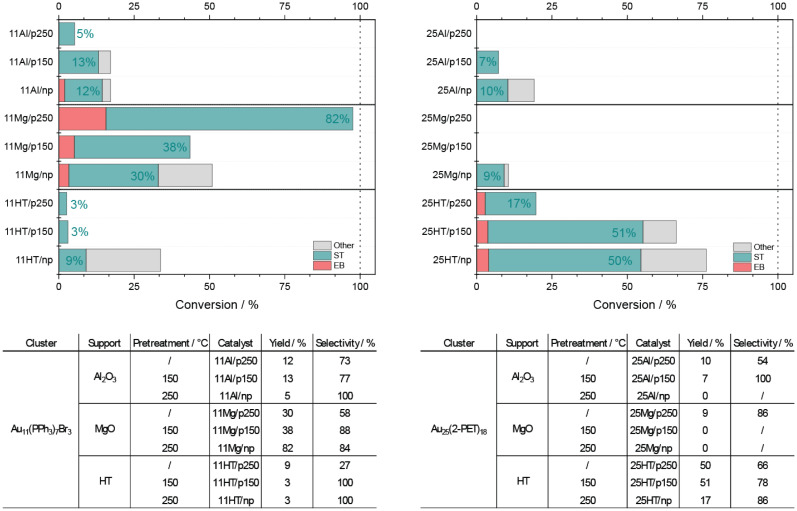
Conversion of phenylacetylene is represented by the total height of the bar. The different colors represent the selectivity of the catalysts toward styrene (ST), ethylbenzen (EB) and other side-products (other). The tables list the yield and selectivity towards styrene of each catalyst.

The catalytic activity is shown in Fig. 2, where significant variations in the performance of the nanoclusters were observed, influenced by their support, ligands and pretreatment conditions. Among the supported Au_11_ nanoclusters, the 11Mg exhibited the highest activity. Interestingly, the catalyst maintained consistent conversions of around 50% with and without pre-treatment at 150 °C. However, when pretreated at 250 °C, the conversion increased to 98%. In contrast, the other Au_11_ catalysts reached a maximum conversion of only 36%.

The data therefore suggest that the reaction is favoured by the basicity of the MgO support, consistent with previous studies suggesting that basic sites facilitate the cleavage of H_2_.^25^ In addition, the increase in catalytic activity following pretreatment at 250 °C, where most of the ligand shell is removed, exposing the gold core atoms and creating new active sites.

The selectivity of each catalyst is also depicted by the colors of the bars. While the catalytic activity increases after pretreatment at 250 °C, selectivity toward the desired styrene (ST) decreases, with more ethylbenzene (EB) being formed. This indicates the need to balance activity and selectivity. The apparent decrease in selectivity could be due to the reaction time; with near-complete conversion, it is likely that all phenylacetylene is converted to ST, after which the next reaction step is the reduction of ST to EB, thereby lowering selectivity. The grey area in the Fig. 2, representing other products, may be attributed to the polymerization of ST, formation of other oligomers, or potential reactions between the substrate and the ligands.

A different trend emerges for the supported Au_25_ nanoclusters. Here, the 25HT variant exhibits the highest activity and selectivity, as illustrated in Fig. 2, with the yield toward ST detailed in Fig. 2. Specifically, the 25HT/np catalyst achieves the highest conversion rate, exceeding 75%. However, unlike the Au_11_ catalysts, the pretreatment of Au_25_ nanoclusters appears to have an inverse effect: as the pretreatment temperature increases, activity decreases, but selectivity improves. As a result, despite the higher conversion rate of 25HT/np, the 25HT/p150 catalyst proves more efficient, delivering the same ST yield with fewer by-products. After pretreatment at 250 °C, selectivity reaches its peak, although activity is reduced by half. This trend is consistent across other catalysts in the study.

The observed trend seems to be related to the different ligands protecting the Au_25_ nanocluster. Unlike the phosphine-protected Au_11_, the thiolate-protected Au_25_ shows higher conversion rates without pretreatment, suggesting that the ligands are actively involved in the catalytic process. This difference could be due to the structural variations between the nanoclusters. In Au_11_ the phosphine ligands are directly bonded to the gold core, whereas in Au_25_ the thiolates are connected *via* staple units. These units have positively charged Au atoms in contrast to the metallic Au in the core. We hypothesise that this difference in electronic structure influences the catalytic behaviour. In addition, the staple arrangement in Au_25_ potentially exposes more of the gold core to hydrogen activation, which may explain the higher activity observed at lower pretreatment temperatures.

The decrease in activity might also be due to the interaction between the exposed gold core and the present PA. Recent work by the Tsukuda group has shown that PA can act as a ligand for gold nanoclusters.^29^ This suggests that if the gold atoms are unprotected of their ligands or even some staple units during pretreatment, PA could bind to the Au atoms, effectively poisoning the active sites. Interestingly, PA does not appear to bind to the gold in the Au_11_ nanocluster, suggesting that the structural differences influence ligand affinity.

To further study the stability of the catalyst during the reaction, recyclability tests of the most promising candidate, the 11Mg catalyst, were performed.

To investigate the effects of the ligands and supports independently, reference experiments were performed using pure supports, free ligands and a blank measurement without any catalyst (ESI Fig. 3 and Table 1†). The blank and reference with the support only experiments showed a negligible PA decrease concentration with no product formation, within the error range of the analysis, confirming that the catalysis is driven by the interaction between the support and the nanoclusters. In contrast, experiments with the free ligands resulted in a noticeable increase in activity (16% for PPh_3_ and 20% for 2-PET) without product formation. This suggests that the interactions between the ligands and the substrate could contribute to the attributed formation of some by-products or even to the formation of new compounds.

### 2.2 XAFS analysis

In order to gain insight into the structural dynamics of the clusters influenced by the support and the reaction conditions, XAFS measurements were performed on the Au-L_3_ and S-K edges of the two more active catalysts. The other XANES studies on the catalysts are given in the ESI.†

#### 2.2.1 XANES analysis

**Fig. 3:**
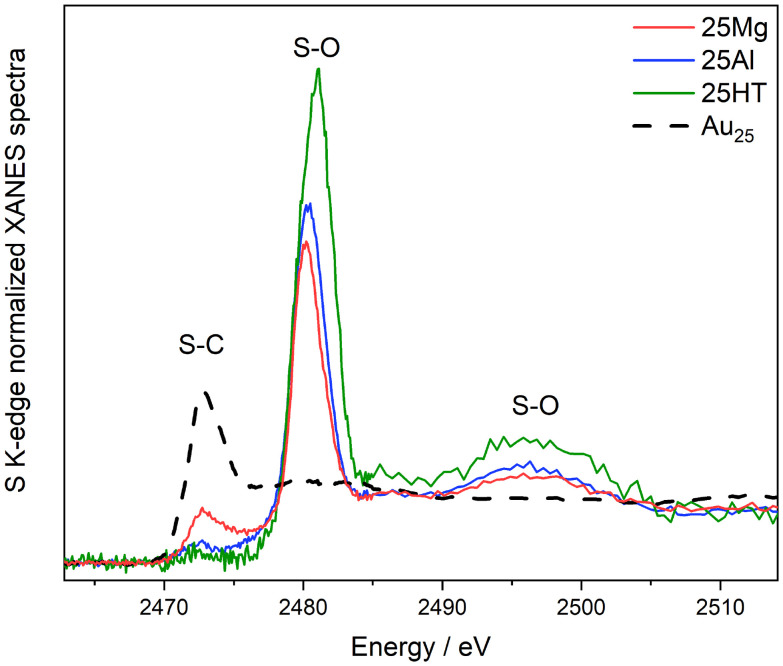
XANES region of the XAFS spectra measured on the S-K edge, of the Au_25_ catalysts. Unsupported Au_25_ nanoclusters were measured and plotted as reference.

The interaction between the thiolated ligands and the staple units was investigated using S K-edge XANES spectroscopy, as illustrated in Fig. 3. Comparing the spectra of the pure cluster with those of the supported cluster reveals a strong interaction between the ligand shell and the support. This is indicated by the decrease in the S–C band around 2472 eV and the emergence of new bands around 2480 eV and 2490 eV, which are attributed to S–O species. The intensity of these new bands varies depending on the type of oxide material, supporting the hypothesis that the interaction between the ligands and the support is influenced by the support's properties. These findings also corroborate our earlier results, which suggested ligand migration from thiolated clusters to the oxide material surfaces.^30,31^

**Fig. 4:**
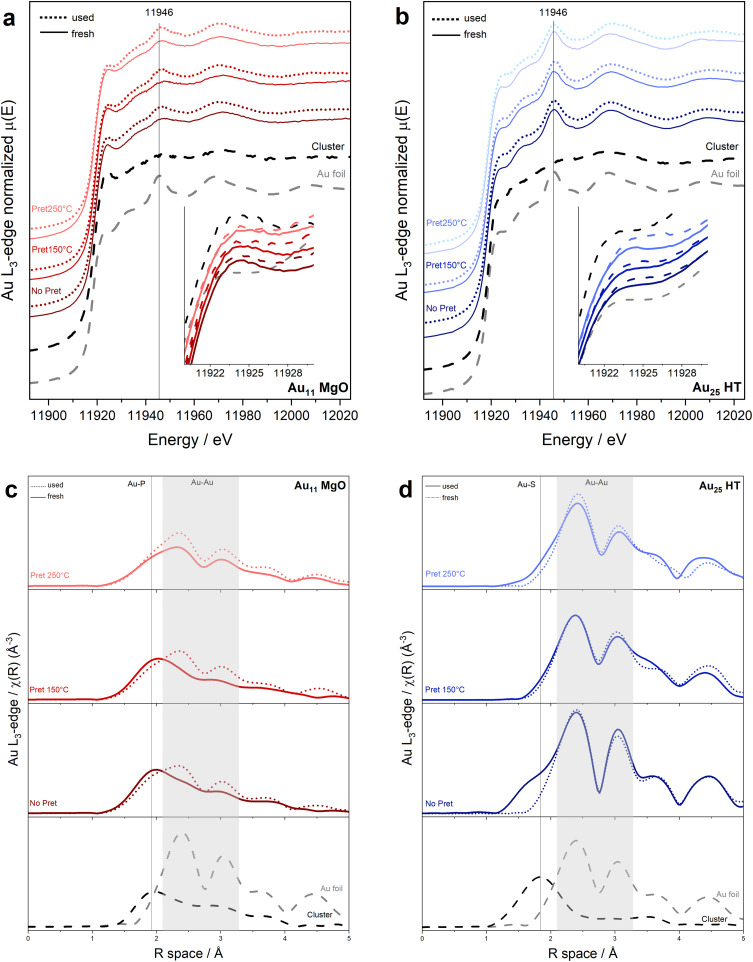
XAFS analysis results of the two catalysts showing better catalytic activity, 11Mg and 25HT, (a) and (b) Au L_3_-edge XANES; (c) and (d) *R*-space EXAFS spectra of the catalysts fresh, pretreated and after reaction.

Fig. 4 presents the XANES spectra of the both cluster catalysts. At the Au L_3_ edge, the spectra of the supported catalysts are compared with those of the pure unsupported cluster and Au foil as references. The results reveal no significant differences between the cluster before and after support. However, the pretreatment leads to a slight decrease in the intensity of the white line (feature at 11 924 eV), which is associated with an increase in the population of metallic Au atoms. This observation also aligns with the ligand loss. The changes are notably more pronounced in the Au_25_ compared to the Au_11_ cluster catalysts. Additionally, the spectra of the catalysts after the reaction show no significant differences or changes in the oxidation state.

When analyzing the XANES of the 11Mg catalysts after the second reaction cycle (Fig. 5 in ESI†), no significant changes can be seen in any of the features, indicating no drastic change in the electronic properties of the clusters during the reaction.

#### 2.2.2 EXAFS analysis

**Fig. 5:**
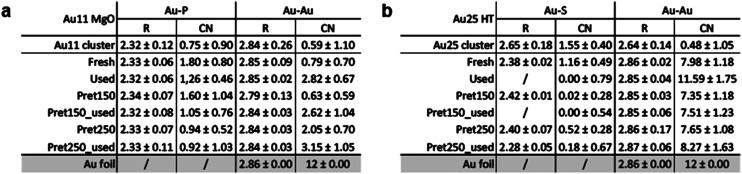
XAFS analysis results of the two catalysts showing better catalytic activity, 11Mg and 25HT, (a) and (b) Au L_3_-edge EXAFS fitting results.

The previous observations were further corroborated by the EXAFS fitting results presented in Fig. 4(c and d) and the corresponding Tables in Fig. 5. The well-resolved cluster structure allows for atomic-level insights, focusing on changes in the coordination number (CN) and average bond distances (*R*) for Au–S or Au–P bonds, depending on the ligand type. These changes reveal the evolution of the ligands, while alterations in the Au–Au bonds provide information about the core structure. (Details of the fitting process can be found in the ESI.†)

Referring to the *R*-space graphs (Fig. 4) and corresponding fitting results (tables in Fig. 5a and b), the phosphine ligands (Fig. 4c) require higher temperatures for removal, as indicated by the persistent peak associated with the Au–P bond and the slight decrease in the CNAu–P values. A notable decrease in CNAu–P occurs after pretreatment at 250 °C, correlating with the catalytic activity observed in Fig. 2, where significant activity is achieved just after this pretreatment. This behavior is consistent with prior studies on temperature-dependent ligand removal in gold nanoclusters, which demonstrate that phosphine ligands are more thermally stable than thiolate ligands, requiring higher temperatures for complete removal.

The removal of ligands exposes more gold surface atoms, likely enhancing catalytic performance by increasing the availability of active sites and potentially generating new ones.^32,33^

Regarding the cyclic experiments, the analysis of the *R*-space, which can be found in the ESI,† shows an increase in the intensity of the bands corresponding to Au–Au bonds, while maintaining a small shoulder in place of Au–P. Furthermore, it can be seen that all clusters, regardless of pre-treatment temperature, have the same *R*-space.

However, the Au11-based catalysts pretreated at 250 °C exhibit markedly different catalytic behavior depending on the support material. This indicates that catalytic activity is influenced not only by the accessibility of exposed gold atoms but also by their interaction with the support surface. In the case of Au11 on MgO, the enhanced catalytic activity can be attributed to the synergistic effect between Au and MgO. Bond *et al.* demonstrated that this combination enhances hydrogenation reactions by promoting both hydrogen dissociation and chemisorption. The basic nature of MgO likely contributes to this effect by modulating the electronic properties of the gold particles, further enhancing their catalytic activity.^15^

Stability of the Au11 core cluster structure is evidenced by the EXAFS results. Upon comparing the catalysts before and after the reaction, a slight increase in the Au–Au coordination number (CNAu–Au) is observed, while the Au–Au bond distance (*R*Au–Au) remains constant. This increase is likely due to the removal of some phosphine ligands under reaction conditions, as suggested by the decrease in the Au–P coordination number (CNAu–P). The cluster's ability to maintain its core structure while undergoing minor surface reorganization, driven by ligand dynamics, may even enhance its catalytic activity by striking a balance between stability and structural flexibility.

In the case of thiolate-protected Au25 clusters, different trends in structure and ligand dynamics are observed. The distinct structural features of these clusters, such as the presence of staple motifs (–S–Au–S–Au–S–) surrounding the gold core, must be considered. Pretreatment of these clusters leads to the near-complete removal of Au–S bonds (as indicated by the decrease in CNAu–S in Fig. 4d and table on the bottom right). This is also consistent with the S K-edge results (Fig. 3), which reveal a strong interaction with the support and the formation of SO_*x*_ species. Since the staples surround the gold core, they are the part that interacts most strongly with the support, influencing both the structural stability and reactivity of the clusters. Without pretreatment, a higher CNAu–Au is observed, likely due to sintering of the gold clusters. However, with mild pretreatment at 150 °C, the catalytic activity remains high, and the main cluster structure shows greater stability, with minimal change in CNAu–Au, in agreement with previous works using even more reactive supports.^28,32^ When higher pretreatment temperatures are applied, an increase in CNAu–Au is observed, accompanied by a drop in catalytic activity.

In this case, we are working with hydrotalcite materials (MgAlO) that exhibit intermediate acid–base properties and basic sites on a high surface area. Upon fragmentation of the staple units, the gold core remains intact, while Au atoms from the staples become dispersed across the support. It can be hypothesized that, without pretreatment, the core and staple units exhibit greater mobility under reaction conditions, leading to a more random distribution of gold species. In contrast, pretreatment stabilizes the Au atoms on the support, including those from the staple units, which retain a partially charged state that is favorable for hydrogen activation. However, at higher pretreatment temperatures, these units become more dynamic, interacting with neighboring gold atoms, as reflected in the slight increase in CNAu–Au observed in the EXAFS fitting results.

The balance between stabilization and mobility of gold species on the support, in this case hydrotalcite, is crucial in determining the catalyst's activity and selectivity. This will also explain the completely different catalytic behaviour of the same cluster in different types of oxides materials with different acid–base properties and surface areas. Optimal pretreatment conditions promote the dispersion of gold atoms from the staple units while maintaining enough stability to prevent excessive sintering or agglomeration. The surface properties of the support also play a critical role in stabilizing both Au and S/P atoms, influencing the interactions between the gold species and the support.

Moreover, depending on the nature of the ligand bonding, direct bonding with phosphines or staple motifs in the case of thiolates, the interaction with the support and the structural dynamics vary, affecting the catalyst's reactivity. For instance, in the case of Au11 clusters, the complete removal of ligands is required to create partially charged Au species due to interactions with the support. However, the choice of support material is equally important to facilitate this interaction and charge state. In contrast, for staple motifs, the partially charged gold at the staple sites seems to be one of the most reactive species, as suggested by theoretical calculations from C. Liu *et al.*^34^ Therefore, the interaction between the staples and the support, which is influenced by the properties of the support material, determines the dynamics of the gold particles and, consequently, their reactivity.

## 3 Conclusions

This study elucidates the crucial roles of ligands and support materials in shaping the catalytic behavior of Au11 and Au25 nanoclusters in the selective hydrogenation of phenylacetylene to styrene. The type of ligand—phosphine in Au11 and thiolate in Au25—has a profound effect on catalytic performance. For Au11, the complete removal of phosphine ligands is necessary to reveal active sites and form partially charged Au species that interact effectively with the support. Conversely, thiolate-protected Au25 clusters benefit from the reactivity of their staple motifs, where partially charged gold atoms are active even before ligand removal.

The interaction between the acid–base properties of the support and the ligand type is pivotal in modulating catalytic performance. MgO, with its basic sites, enhances Au11 activity by promoting hydrogen dissociation and stabilizing partially charged Au species. On the other hand, hydrotalcite, with intermediate acid–base properties, provides optimal results for Au25, stabilizing staple motifs and maintaining catalytic selectivity at lower pretreatment temperatures.

The study demonstrates that the nature of ligand bonding and its interaction with the support material critically affects catalyst reactivity. For Au11, ligand removal is essential for creating reactive Au species that interact with the support, while thiolate-protected Au25 benefits from the inherent reactivity of staple motifs. The support's properties are key to influencing these interactions and the dynamics of the gold particles.

In summary, achieving an optimal balance between nanocluster stabilisation and gold species mobility is essential to maximise catalytic performance. This work highlights the importance of fine-tuning ligand removal and support interactions to optimise both activity and selectivity, and provides valuable insights for the rational design of gold-based nanocluster catalysts for selective hydrogenation reactions.

## 4 Experimental

The nanoclusters were synthesized according to previous experience and characterized by Ultra-Visible (UV-Vis) spectroscopy and Matrix Assisted Laser Desorption Ionisation Mass Spectrometry (MALDI-MS), with the details found in the ESI.† They were supported on the metal oxides by dissolving and mixing with toluene or ethanol, further described in the ESI.† Characterization of the supports was done by X-ray powder diffraction (XRD). The catalysts were calcined at 150 °C and 250 °C in a calcination oven. The catalytic tests were conducted in liquid phase, in an autoclave. Structural evolution of the clusters was studied by X-ray Absorption Fine Structure Spectroscopy (XAFS). Details of all procedures can be found in the ESI.†

## Author contributions

Conceptualisation – N.B., A.P., F.R., methodology – N.B., C.M, investigation – N.B., R.B., A.L., N.M, S.S., C.M., formal analysis – N.B., C.M., R.B., writing (original draft) – N.B., R.B., writing (review and editing) all, resources – N.B., supervision – N.B.

## Data availability

All data and analyses supporting this article are available on the TU Wien repository (reposiTUm) at [URL – https://researchdata.tuwien.at/uploads/45pt2-ygs20].

## Conflicts of interest

There are no conflicts to declare.

## Supplementary Material

NR-017-D4NR03865G-s001
