# Supplementary material for: Synergistic effect of ligand–cluster structure and support in gold nanocluster catalysts for selective hydrogenation of alkynes
Source: Nanoscale. 2025 Jan 14;17(9):5098–105. doi: 10.1039/d4nr03865g (PMC11753004; doi:10.1039/d4nr03865g)
Supplement: NR-017-D4NR03865G-s001 [file NR-017-D4NR03865G-s001.pdf]

Supplementary Information (SI) for Nanoscale.
This journal is © The Royal Society of Chemistry 2025

# Synergistic Effect of Ligand-Cluster Structure and Support in Gold Nanocluster Catalysts for Selective Hydrogenation of Alkynes

Rareș Banu,[a] Adea Loxha,[a] Nicole Müller,[b] Stylianos Spyroglou,[b] Egon Erwin Rosenberg,[c] A. Eduardo Palomares,[d] Fernando Rey,[d] Carlo Marini,[e] Noelia Barrabés*[a]

[a] *Institue of Materials Chemistry, TU Wien, Getreidemarkt 9/E165, 1060 Vienna, Austria*
[b] *Institute of Chemical, Environmental and Bioscience Engineering, TU Wien, Getreidemarkt 9/E166, 1060 Vienna, Austria*
[c] *Institute of Chemical Technologies and Analytics, TU Wien, Getreidemarkt 9/E164, 1060 Vienna, Austria*
[d] *Instituto de Tecnología Química, Universitat Politécnica de Valéncia - Consejo Superior de Investigaciones Científicas (UPV-CSIC), Valencia, Spain*
[e] *ALBA Synchrotron Light Facility, Carrer de la Llum 2-26, 08290, Cerdanyola del Valles, Barcelona, Spain*

## 1 Materials

The reagents, solvents and other consumables used in the preparation of nanoclusters were obtained from commercial suppliers. Any steps involving aqueous solutions were performed using ultrapure Milli-Q $H_2O$ (18.2 MΩ · cm at 25 °C).

Hydrogen tetrachloroaurate trihydrate, $HAuCl_4 \cdot 3\ H_2O$ (≥49.0 % Au basis), sodium tetrahydroborate, $NaBH_4$ (98 %) and triphenylphosphine (≥99 %) were obtained from Alfa Aesar. Tetraoctylammonium bromide, TOABr (≥98 %) was ordered from TCI Chemicals. 2-phenylethanethiol (98 %) and phenylacetylene (98 %) were purchased from Sigma Aldrich. Pyridine (ACS, Reag. Ph Eur) was aquired from Supelco. HPLC grade ethanol and toluene were purchased from Carl Roth. Milli-Q water (resistivity of 18.2 MΩ · cm at 25 °C) was used in the synthesis procedure. All solvents used (dichloromethane, methanol, tetrahydrofuran, toluene, etc.) were at least of synthesis grade and used without further purification. Bio Beads S-X1 support (Bio-Rad) was used for size exclusion chromatography (SEC).

## 2 Nanocluster Synthesis

### 2.1 $Au_{11}(PPh_3)_7Br_3$.

The cluster was prepared according to a previously reported protocol by Truttmann *et al.*[1] A solution of $HAuCl_4 \cdot 3\ H_2O$ (1 eq., 501 mg, 1.27 mmol) and TOAB (1.2 eq., 833 mg, 1.52 mmol) were dissolved in 50 mL THF in a round bottom flask, forming an orange solution. Subsequently, triphenylphosphine (5 equiv., 1162 mg, 6.34 mmol) was added, whereupon a colorless mixture was obtained. The solution was allowed to stir for 1 h at room temperature, followed by the addition of $NaBH_4$ (10 equiv., 474 mg, 12.54 mmol) dissolved in 10 mL cold Mili-Q water.

The reaction mixture was stirred for 48 hours, resulting in a dark brown precipitate. After evaporation of the solvent under reduced pressure, the remaining solid was washed with a 1:1 mixture of MeOH:H2O. Then, the by-products were extracted with THF, toluene, and a 2:1 mixture of hexane:EtOH, respectively. Each solution was analyzed *via* UV-Vis. The $Au_{11}$ cluster was extracted with DCM, showing two prominent bands. Upon evaporation of the solvent an orange solid was obtained. The product was characterized by UV-Vis and the spectrum is displayed in the following.

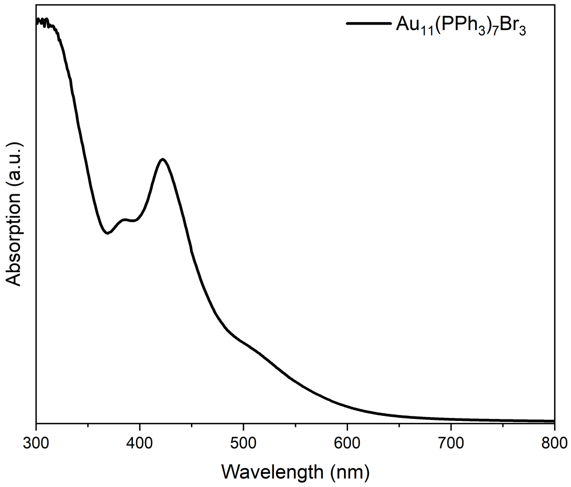


Figure 1: UV-Vis spectrum of the pure $Au_{11}(PPh_3)_7Br_3$ nanocluster.

### 2.2 $Au_{25}(SC_2H_4Ph)_{18}$ and $[Au_{25}(SC_2H_4Ph)_{18}]^-TOA^+$.

The cluster was synthesized following previous experience.[2] To begin with, TOABr (1.2 eq., 833 mg, 1.52 mmol) and $HAuCl_4 \cdot 3\ H_2O$ (1 eq., 500 mg, 1.27 mmol) were dissolved in 50 mL THF, after which 850 $\mu$L of 2-PET were added, whereupon the color of the orange solution faded out after stirring for 1 h. Then, 480 mg of $NaBH_4$ in 10 mL cold Mili-Q water were added. The mixture was stirred at room temperature for 4 days. Upon evaporation of THF, the precipitate was repeatedly washed with a 1:1 mixture of water in methanol and then with pure methanol. The crude product was extracted with acetone and purified *via* SEC, yielding a black solid. The product was characterized by UV-Vis and MALDI-MS and the spectra are displayed in the following Figure.

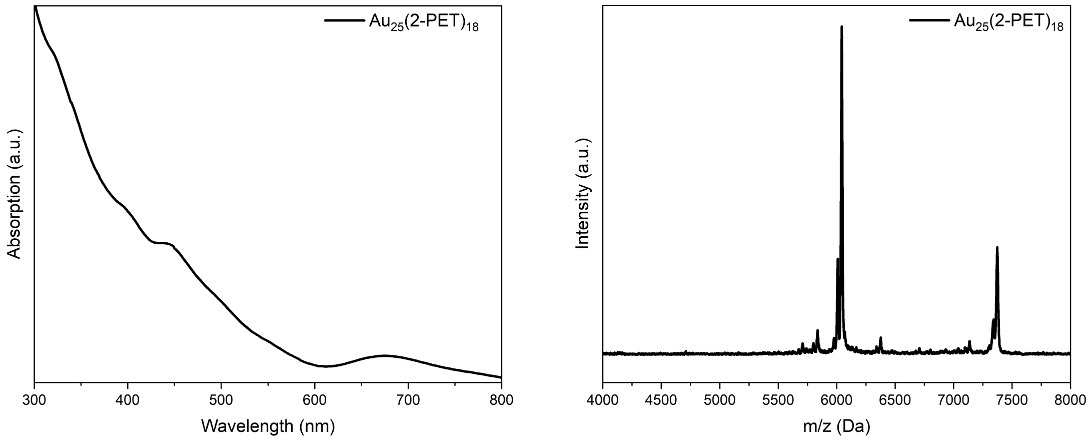


(a) UV-Vis spectrum of the pure $Au_{25}$(2-PET)$_{18}$ nanocluster.

(b) MALDI-MS spectrum of the pure $Au_{25}$(2-PET)$_{18}$ nanocluster.

Figure 2: Characterization of the $Au_{25}$(2-PET)$_{18}$ nanocluster.

## 3 Preparation of supports.

The $\alpha$-$Al_2O_3$ was procured commercially from abcr GmbH. The hydrotalcite was synthesized according to literature[3] by another member of the group. To this end, the co-precipitation method was applied. A mixture of $Mg(NO_3)_2$ and $Al(NO_3)_3$ of acidic pH was mixed with a basic one of NaOH and $NaNO_3$, whereby the reactants were chosen such as to obtain a molar ration of 4 of Mg:Al. The solutions were mixed drop-wise

at room temperature and standard pressure, using an infusion pump with a flow of 1 mL/min.Following this, the solution was aged at 60 °C for 24 h, after which the solid was filtered and washed with water until a pH of 7 was reached. Finally, the HT was dried at 100 °C for 24 h. The MgO was also prepared by another member of the group, according to previous experience[3]. In this case, 25 g of $(MgCO_3)_4Mg(OH)_2$ were suspended in 750 mL of water and stirred at 80 °C for 30 min, after which the solid was filtered and dried at 90 °C for 24 h, followed by calcining at 450 °C for 2 h.

# 4 Preparation of supported nanoclusters.

Both $Au_{25}$ and $Au_{11}$ were supported on all three metal oxides, with a metal loading of 0.05 wt% following the same protocol. The appropriate amount of nanocluster was dissolved in HPLC grade toluene or ethanol respectively, and mixed with a suspension of the metal oxide in the same solvent. The mixture was then stirred for 24 h at room temperature, after which the now clear solvent was slowly removed under reduced pressure. The obtained powder was dried in an oven at 80 °C for 1 h, after which parts of it were pretreated by calcining in an oven at 150 °C and 250 °C for 1 h, heating at a rate of 10 °C/min.

# 5 Catalytic tests.

The catalytic tests were performed in an autoclave, at 8 bar $H_2$ and 100 °C. The reactor was purged three times with hydrogen before the final filling. 2 mg of catalyst, 0.08 mL pyridine, 0.11 mL phenylacetylene and 2 mL of ethanol were reacted for 24 h at the specified temperature and pressure, in batches of six. The samples were filtered using syringe filters, and the solution was analyzed by gas chromatography (GC). For the recyclability experiments, the reaction solution was separated from the catalysts, the catalysts washed 3 times with ethanol, dried under nitrogen and new reactants were added, after which the new reaction cycle was started.

The quantification was done by external calibration on GC, on a Schimadzu GC-2014, equipped with a Flame Ionization Detector (FID) and a HP-5 column of 30 m length, 0.25 $\mu$m film thickness and 0.32 mm column ID. For each injection, the syringe was rinsed three times with solvent, followed by rinsing three times with sample and after the injection, another three solvent rinses. 1 $\mu$L of sample was injected for each measurement and each sample was measured three times. The initial temperature of the GC was held at 60 °C for 7 min, followed by heating up to 250 °C with a samp of 50 °C/min and a holding time of 2 min.The oven temperature was 60 °C, the injection chamber was held at 250 °C and he FID at 300 °C. The measurements were done in Split mode, with a split of 18:100, a column flow of 2.49 mL/min and a total flow of 50.1 mL/min.

The peaks were integrated by hand and the the areas converted to concentrations using the linear regression obtained from the external calibration. The conversion ($X$) and selectivity ($S$) were calculated according to the following formulae. The yield was calculated by dividing the selectivity by the conversion.

$$X = \frac{c_{\mathrm{PA},0}}{c_{\mathrm{PA}}} \cdot 100$$

$$S_{\mathrm{i}} = \frac{c_{\mathrm{i}}}{\sum c_{\mathrm{i}}} \cdot 100$$

## 5.1 Blank experiments.

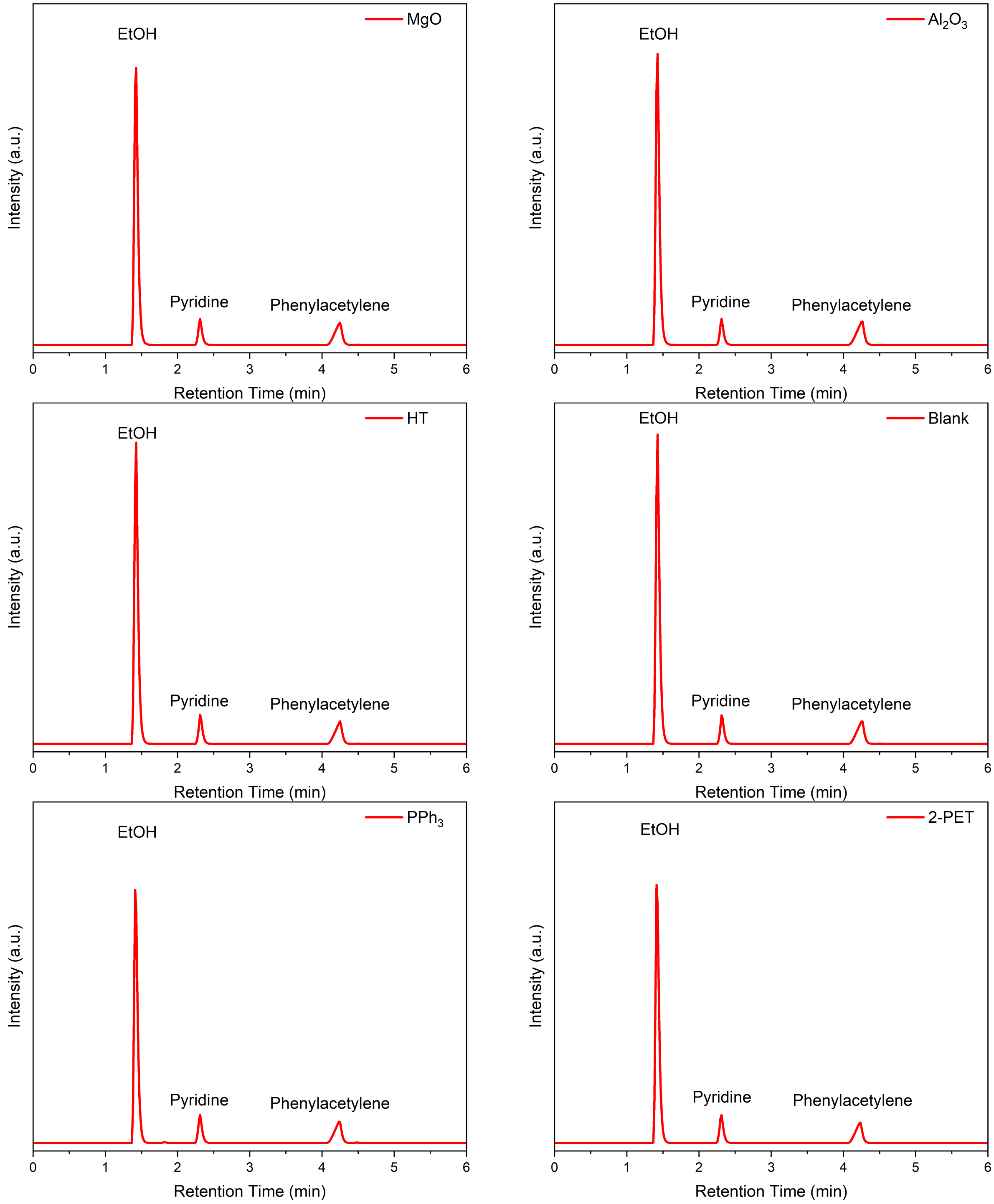


Figure 3: Chromatograms of the blank experiments done in order to gauge the effect of the supports and the ligands on the catalytic activity.

Table 1: Table depicting the results of the blank experiments.

| Sample | Conversion / % | Yield / % | | |
|---|---|---|---|---|
| | | EB | ST | Other |
| Blank | 5 | 2 | 3 | 0 |
| $Al_2O_3$ | 2 | 1 | 1 | 0 |
| HT | 11 | 2 | 9 | 0 |
| MgO | 9 | 2 | 7 | 0 |
| $PPh_3$ | 16 | 0 | 10 | 6 |
| 2-PET | 20 | 1 | 9 | 10 |

## 5.2 Recyclability experiments.

Table 2: Comparison of the catalytic activity of the cluster catalysts between two reaction cycles (yield and selectivity related to styrene. As it can be seen, the catalysts remain active in the second cycle.

| Cluster | Cycle | Pretreatment / C | Catalyst | Yield / % | Selectivity / % |
|---|---|---|---|---|---|
| $Au_{11}(PPh_3)_7Br_3$ | 1st | / | 11Mg/np | 48 | 71 |
| | | 150 | 11Mg/p150 | 64 | 69 |
| | | 250 | 11Mg/p250 | 34 | 91 |
| | 2nd | / | 11Mg/np | 59 | 27 |
| | | 150 | 11Mg/p150 | 56 | 40 |
| | | 250 | 11Mg/p250 | 16 | 56 |

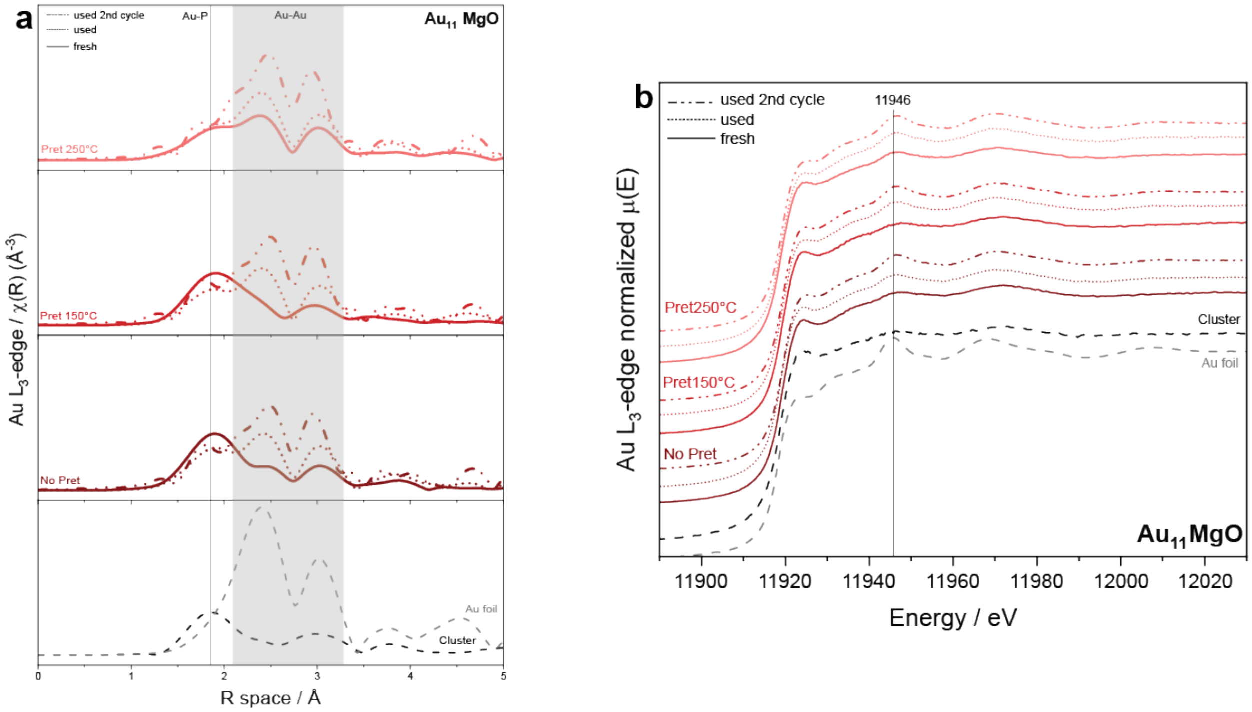


Figure 4: XAFS analysis of the 11Mg catalysts used for the recyclability tests. Panel a shows the R-space and panel b the XANES region. It can be seen, that the structure is retained, between the first and second cycles.

# 6 Characterization

## 6.1 Ultraviolet-Visible Spectroscopy.

UV-Vis spectroscopy was performed on a UV-1600PC spectrometer using cuvettes of 1 cm pathlength. Different solvents (DCM, toluene, THF) were used to dissolve the Au nanoclusters depending on the specific reaction step.

## 6.2 Matrix-assisted Laser Desorption/Ionization (MALDI-MS).

Matrix-assisted laser desorption/ionization mass spectrometry (MALDI-MS) was conducted on a Bruker Ultraflextreme MALDI-TOF instrument equipped with a Nd:YAG laser in linear mode. Each spectrum was obtained by averaging 5000 single shots (split in packets of 500 shots). Spectra were obtained at 10 % laser power. trans-2-[3-(4-tert-Butylphenyl)-2-methyl-2- propenyliden]-malononitrile (DCTB) was used as matrix. Sample and matrix solutions were prepared in toluene.

## 6.3 X-Ray diffraction (XRD).

Powder X-ray diffraction (XRD) was performed using a PANalytical X'Pert powder diffractometer in Bragg-Brentano geometry. The diffractometer was equipped with a Cu LFF X-ray tube operated at 45 kV and 40 mA (Cu $K_\alpha$), a BBHD mirror, and a Malvern PANalytical MPD Pro (PW3050/60 goniometer) with 200 mm goniometer radius. The $2\theta$ ranges were set between 4,99 to 128° with a step size of 0.02°.

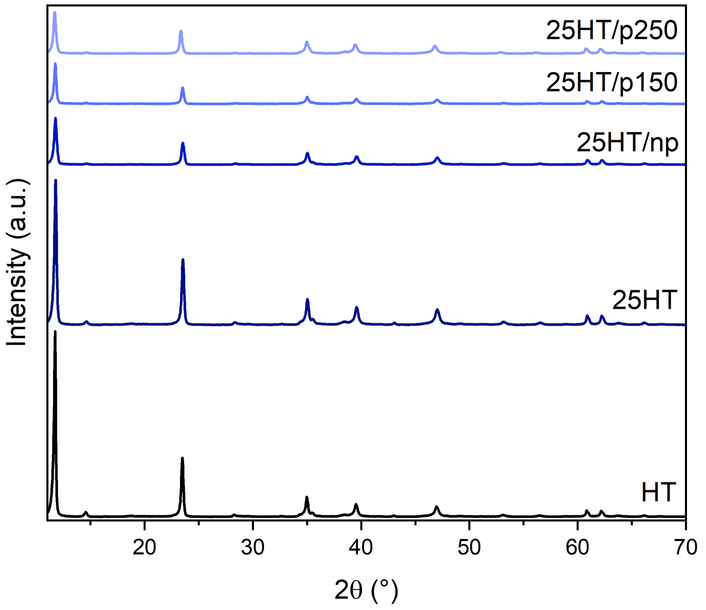


Figure 5: XRD measurement of the 25HT catalysts, with the bare support as reference, made to ensure that the structure of the hydrotalcite was retained after pretreatment and reaction.

# 7 X-Ray Absorption Fine Structure (XAFS) Spectroscopy

XAFS measurements were performed at the CLAESS Beamline at Alba Synchrotron in fluorescence mode (Au-$L_3$ edge and S-K edge) in the beamline's sample holder for static measurements. The catalysts were pressed into pellets and secured by kapton tape.

The synchrotron radiation emitted by a wiggler source was monochromatized using a double crystal Si(311) monochromator. The rejection of higher harmonics was done by choosing proper angles and coatings of the collimating and focusing mirrors. XAS measurements were performed in fluorescence continuous mode using a multielement silicon drift detector with Xspress3 electronics. Slit gap in front of I0 ionization chamber have been set to different values in order to guarantee the correct deadtime values depending on the sample measured. Energy scale at Au-$L_3$ and S-K edges have been previously calibrated by measuring the Au and S foils respectively.

The raw spectra have been processed according to standard methods. The have been normalized by approximating pre-edge and post-edge backgrounds are low-order polynomial curves. The corresponding EXAFS signal has been then extracted, k-squared weighted, and Fourier transformed (FT). The fresh, unpretreated samples have been used as starting points for all EXAFS modelling, providing the theoretical phases and amplitudes of the scattering paths by means of self-consistent ab-initio calculations performed with FEFFlite code[4]. Furthermore, in order to get more conclusive structural parameters from the EXAFS, all spectra were fitted on the Au-$L_3$ edge using a personalized code based on IFEFFIT[5].

Two two shell models were considered for fitting the data (with Au-Au and Au-S or Au-P contributions). As fitting parameters, the coordination numbers CN $N_{\text{Au-Au}}$, $N_{\text{Au-S}}$ and $N_{\text{Au-P}}$, as well as three correction factors for the inter-atomic distances $\Delta R_{\text{Au-Au}}$, $\Delta R_{\text{Au-S}}$ and $\Delta R_{\text{Au-P}}$ were used. Furthermore, three disorder parameters were used, $\sigma^2_{\text{Au-Au}}$, $\sigma^2_{\text{Au-S}}$, $\sigma^2_{\text{Au-P}}$. The following table lists the fitting parameters for both systems. The figures corresponding to the fitting are shown in the following.

Table 3: Fitting parameters for the EXAFS spectra.

| amp | Enot(Au) | Enot(Au-S) | Enot(Au-P) | $\sigma^2_{\text{Au-Au}}$ | $\sigma^2_{\text{Au-S}}$ | $\sigma^2_{\text{Au-P}}$ |
|---|---|---|---|---|---|---|
| 0.92 ± 0.22 | 3.73 ± 1.09 | 2.64 ± 1.62 | 8.33 ± 3.63 | 0.009 ± 0.002 | 0.005 ± 0.001 | 0.013 ± 0.003 |

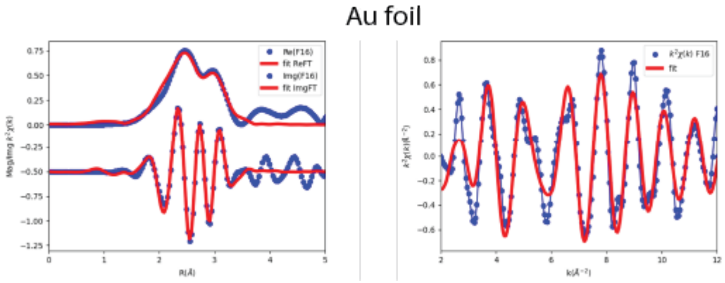


Figure 6: EXAFS fitting of the Au foil.

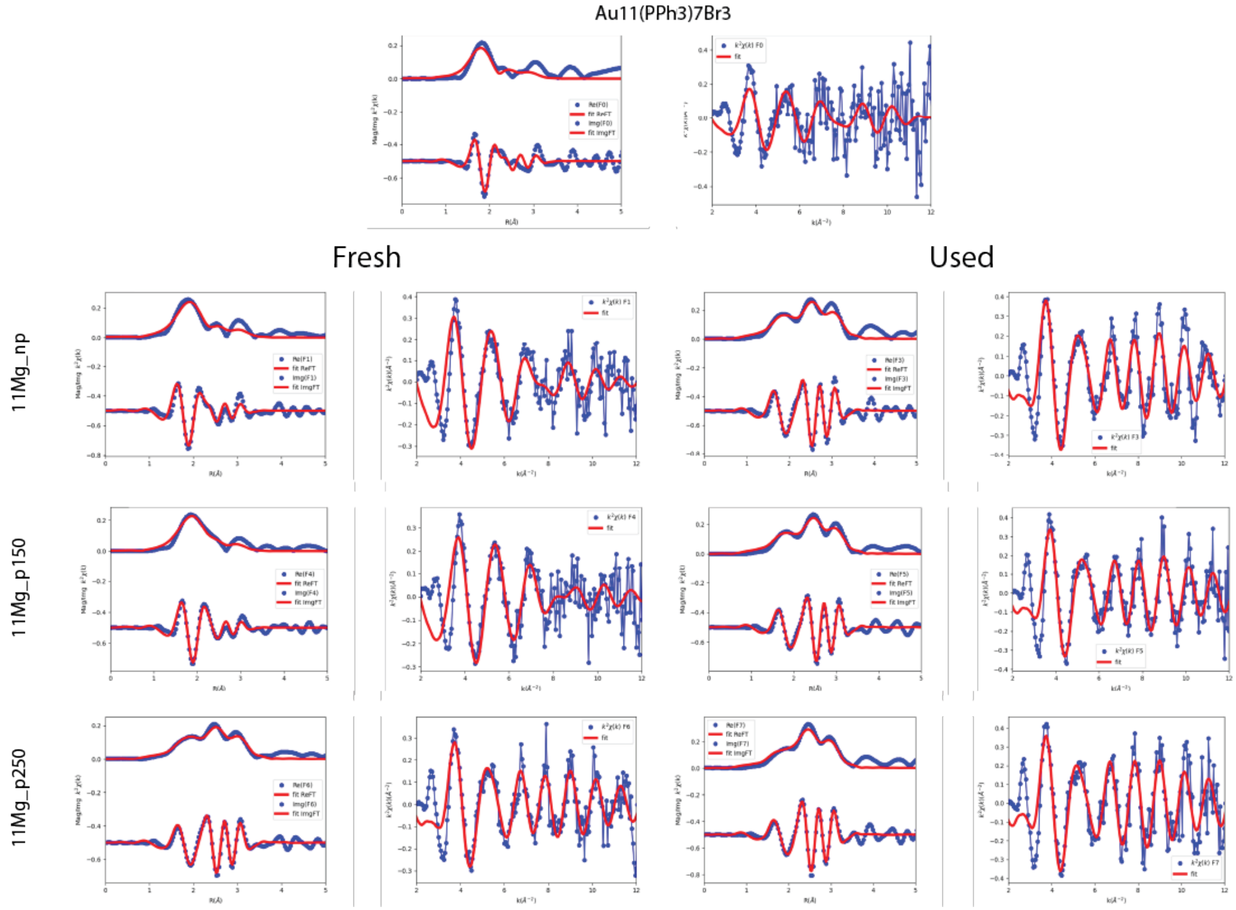


Figure 7: EXAFS fitting of the 11MgO catalysts.

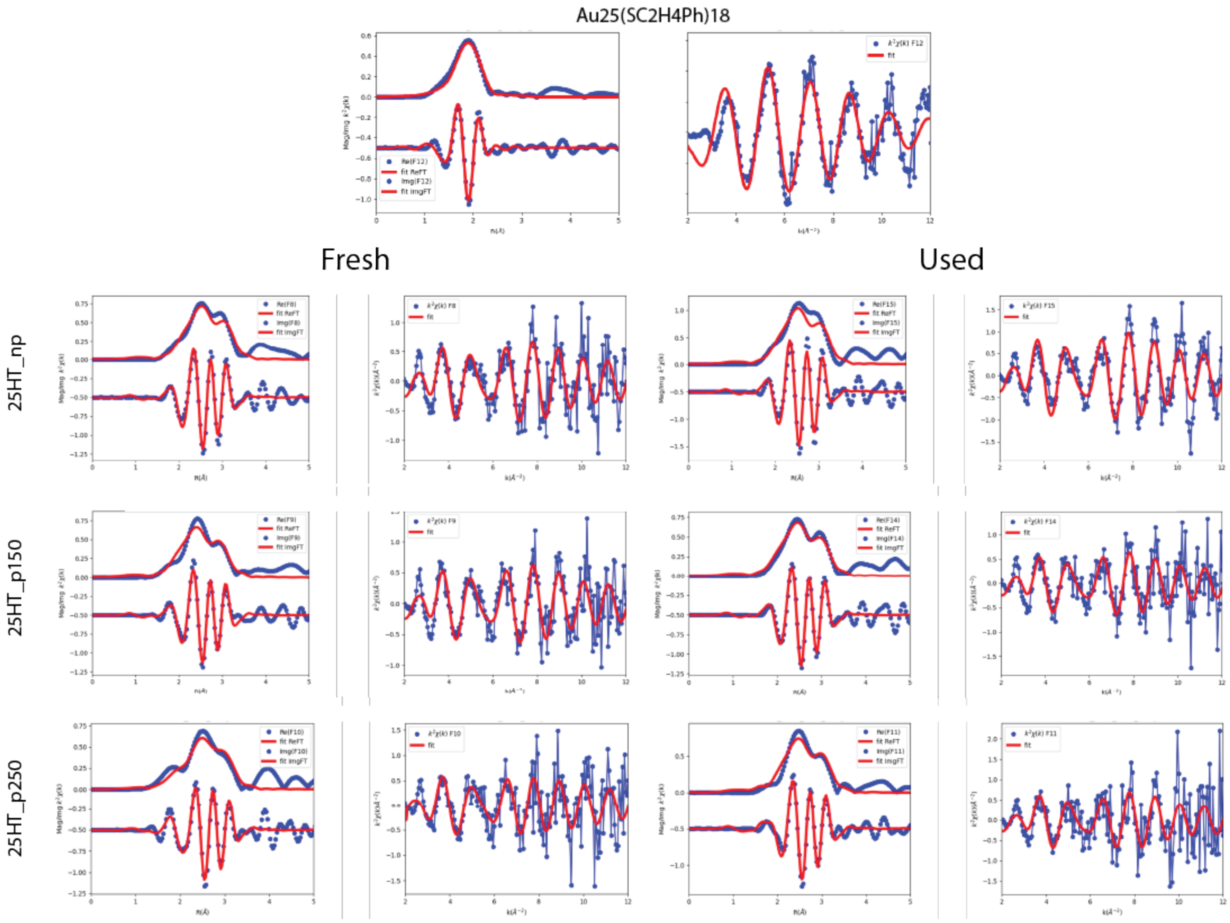


Figure 8: EXAFS fitting of the 25HT catalysts.

The XANES of the 11HT, 11Al, 25Mg and 25Al catalysts are displayed in the following figures.

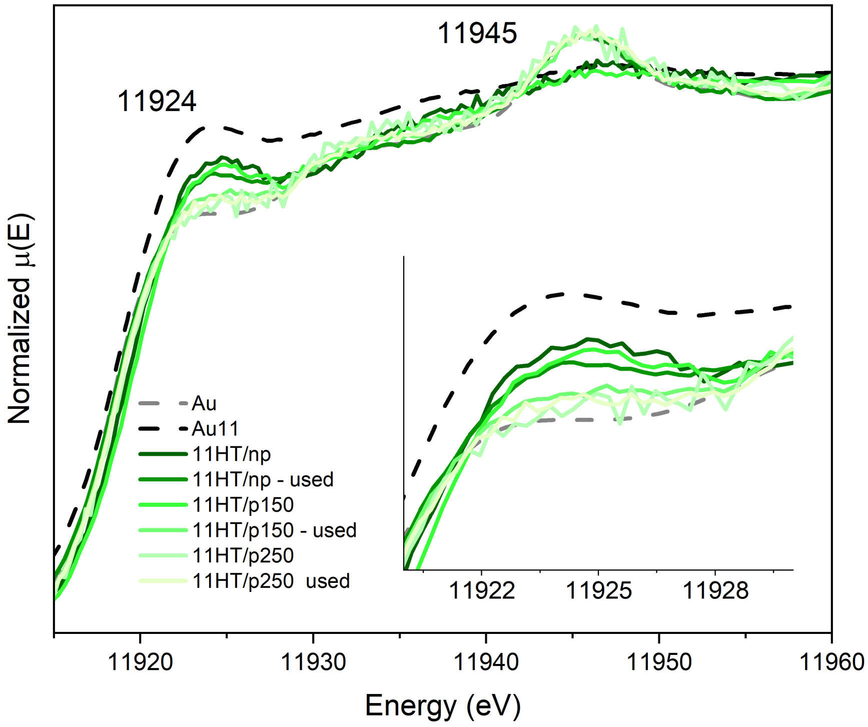


Figure 9: 11HT XANES

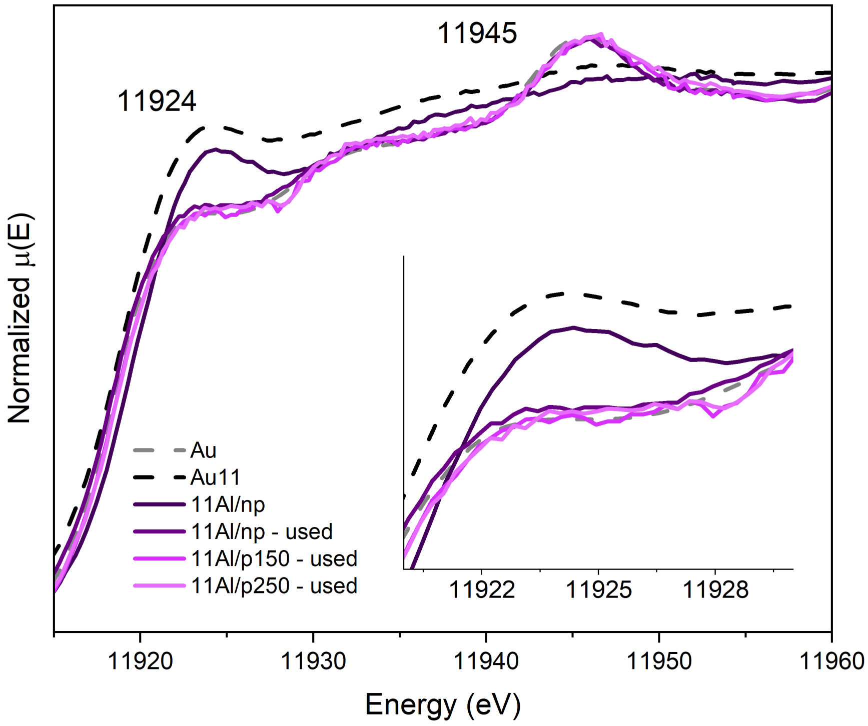


Figure 10: 11Al XANES

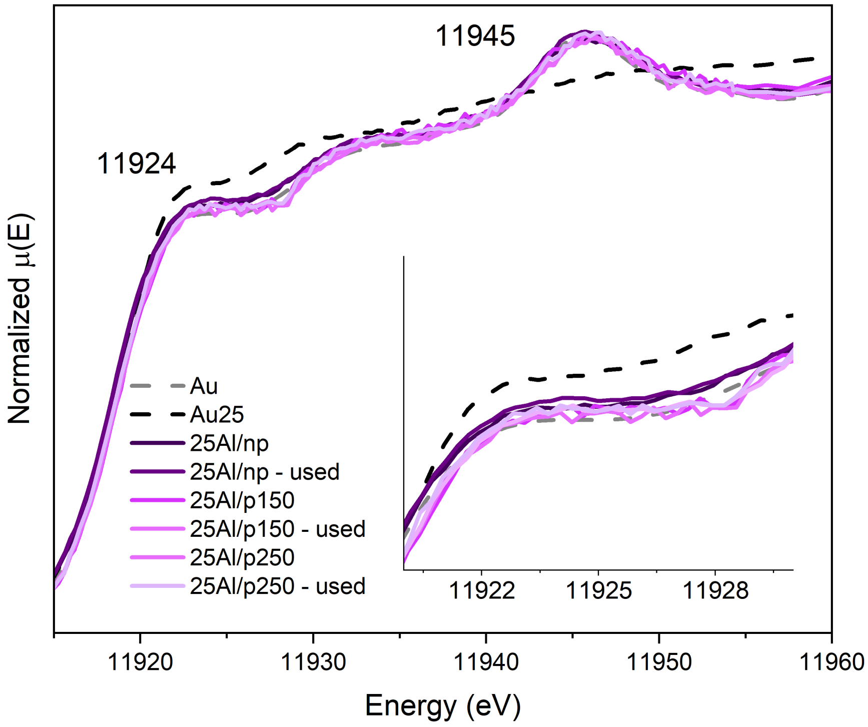


Figure 11: 25Al XANES

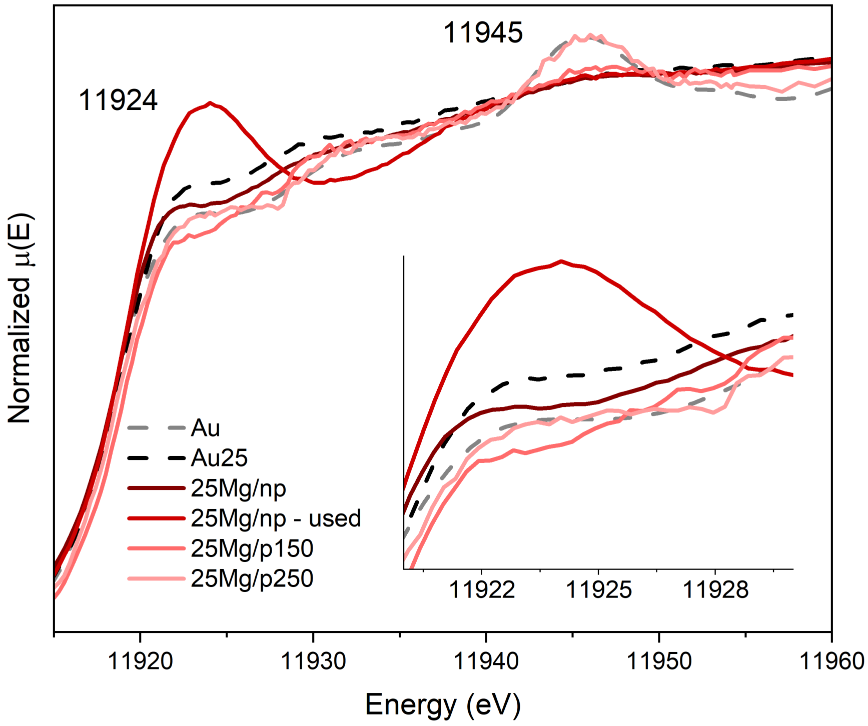


Figure 12: 25Mg XANES

# References


[1] V. Truttmann, C. Herzig, I. Illes, A. Limbeck, E. Pittenauer, M. Stöger-Pollach, G. Allmaier, T. Bürgi, N. Barrabés and G. Rupprechter, *Nanoscale*, 2020, **12**, 12809–12816.

[2] V. Truttmann, A. Loxha, R. Banu, E. Pittenauer, S. Malola, M. F. Matus, Y. Wang, E. A. Ploetz, G. Rupprechter, T. Bürgi, H. Häkkinen, C. Aikens and N. Barrabés, *ACS Nano*, 2023, **17**, 20376–20386.

[3] I. López-Hernández, V. Truttmann, N. Barrabés, G. Rupprechter, F. Rey, J. Mengual and A. Palomares, *Catalysis Today*, 2022, **394–396**, 34–40.

[4] J. J. Rehr, J. J. Kas, F. D. Vila, M. P. Prange and K. Jorissen, *Physical Chemistry Chemical Physics*, 2010, **12**, 5503.

[5] M. Newville, *Journal of Synchrotron Radiation*, 2001, **8**, 322–324.
